# Kuwanon V Inhibits Proliferation, Promotes Cell Survival and Increases Neurogenesis of Neural Stem Cells

**DOI:** 10.1371/journal.pone.0118188

**Published:** 2015-02-23

**Authors:** Sun-Young Kong, Min-Hye Park, Mina Lee, Jae-Ouk Kim, Ha-Rim Lee, Byung Woo Han, Clive N. Svendsen, Sang Hyun Sung, Hyun-Jung Kim

**Affiliations:** 1 Laboratory of Molecular and Stem Cell Pharmacology, College of Pharmacy, Chung-Ang University, Seoul, Republic of Korea; 2 College of Pharmacy and Research Institute of Pharmaceutical Science, Seoul National University, Seoul, Republic of Korea; 3 College of Pharmacy, Sunchon National University, Suncheon, Jeollanam-do, Republic of Korea; 4 Laboratory Science Division, International Vaccine Institute, Seoul, Republic of Korea; 5 Board of Governors Regenerative Medicine Institute, Cedars-Sinai Medical Center, Los Angeles, California, United States; Temple University School of Medicine, UNITED STATES

## Abstract

Neural stem cells (NSCs) have the ability to proliferate and differentiate into neurons and glia. Regulation of NSC fate by small molecules is important for the generation of a certain type of cell. The identification of small molecules that can induce new neurons from NSCs could facilitate regenerative medicine and drug development for neurodegenerative diseases. In this study, we screened natural compounds to identify molecules that are effective on NSC cell fate determination. We found that Kuwanon V (KWV), which was isolated from the mulberry tree (*Morus bombycis*) root, increased neurogenesis in rat NSCs. In addition, during NSC differentiation, KWV increased cell survival and inhibited cell proliferation as shown by 5-bromo-2-deoxyuridine pulse experiments, Ki67 immunostaining and neurosphere forming assays. Interestingly, KWV enhanced neuronal differentiation and decreased NSC proliferation even in the presence of mitogens such as epidermal growth factor and fibroblast growth factor 2. KWV treatment of NSCs reduced the phosphorylation of extracellular signal-regulated kinase 1/2, increased mRNA expression levels of the cyclin-dependent kinase inhibitor p21, down-regulated Notch/Hairy expression levels and up-regulated microRNA miR-9, miR-29a and miR-181a. Taken together, our data suggest that KWV modulates NSC fate to induce neurogenesis, and it may be considered as a new drug candidate that can regenerate or protect neurons in neurodegenerative diseases.

## Introduction

Neural stem cells (NSCs) have proliferative properties and the ability to generate neurons, astrocytes, and oligodendrocytes [1, 2]. NSCs can be cultured as neurospheres. However, neural progenitor cells (NPCs) also exist in the neurospheres. NPCs can’t be expanded for long period of time and show reduced neurogenesis in the later passages [3, 4]. Rat neurospheres contain primarily NPCs and a few NSCs [4]. However, as there are no good markers to distinguish the two cell types, this report refers to the neurospheres as NSCs. Previous studies have identified many intrinsic and extrinsic factors that determine the fate of NSCs, such as age, genetics, epigenetic modifications, hormonal status, mitogens like epidermal growth factor (EGF) and fibroblast growth factor 2 (FGF2), brain-derived neurotrophic factor, neuropeptide Y and retinoic acid [5–7]. Recently, small molecules have also been identified as regulators of NSC fate [8, 9]. Sodium butyrate, a histone deacetylase inhibitor and an anticonvulsant, increases the proliferation and differentiation of NSCs into neurons [10]. Phosphoserine inhibits NSC proliferation and promotes neuronal differentiation and survival by activating metabotropic glutamate receptor 4 [11]. Although the molecular mechanisms underlying regulation of NSC fate are not completely understood, several pathways have been identified that control NSC survival, proliferation and differentiation [12–14]. The mitogens EGF and FGF2, as well as notch activation, play an important role in the maintenance of NSCs by increasing proliferation and inhibiting neuronal differentiation [7, 15–17]. MicroRNAs (miRNAs) are short noncoding RNAs that simultaneously silence hundreds of target genes. They bind to the complementary sequences in 3′-untranlated region of target mRNAs and inhibit translation or induce degradation of target mRNA. miRNAs are recently discovered to control cell fate determination [18].

Because NSCs are present not only in the developing brain but also in specific areas such as the subventricular zone (SVZ) and hippocampus of the adult brain [1, 19], identifying drugs that can protect NSCs or increase neurogenesis from NSCs holds promise for the treatment of neurodegenerative diseases [20]. In many cases, drug screenings have been performed using genetically modified cells or immortalized cells that contain reporter systems [21, 22]. However, the development and advancement of stem cell technology provides cells that are useful in a more accurate molecular screening for novel drugs that can affect neuroprotection or neurogenesis.

Several phytochemicals are known to improve diseases due to their antioxidative and pharmacological effects. The root and bark of mulberry trees (*Morus bombycis* and other plants of the genus *Morus*) have been used as expectorants, diuretics, laxatives, antiphlogistics and antidiabetics [23, 24]. In addition, compounds in the root and bark of these trees have antiviral and antimicrobial, hepatoprotective, hypoglycemic, hypolipidemic, neuroprotective and antioxidant effects [25–30]. Kuwanon V (KWV), a chalcone-derivative phenolic compound isolated from the *M. bombycis* bark [31], has shown inhibitory effects against protein tyrosine phosphatase 1B and hypoxia-inducible factor-1 (HIF-1), suggesting the potential for treating diabetes, obesity and cancers [32, 33]. Furthermore, curcumin isolated from the rhizomes of *Curcuma longa* Linn and casticin isolated from the leaves of *Croton betulaster* Müll. Arg. are reported to modulate the survival, proliferation and differentiation of NPCs [34, 35]. Thus, to discover new phytochemicals that are effective in controlling NSC fates, we screened several natural products including KWV on NSCs.

In this study, we show that KWV protects and increases neuronal differentiation in rat fetal NSCs, even in the presence of EGF and FGF2. KWV treatment reduced the phosphorylation of extracellular signal-regulated kinase 1/2 (ERK1/2), increased mRNA expression levels of the cyclin-dependent kinase inhibitor p21, reduced *notch1* and *hes1* transcription and up-regulated the miRNAs including miR-9, miR-29a and miR-181a. Our findings indicate that KWV is able to modulate NSC fate into neurons, suggesting that it may be used to treat neurodegenerative diseases.

## Materials and Methods

### Plant material collection, extraction and isolation

The *M. bombycis* barks were collected from Nambu Forest of Seoul National University, Baegwoon Mountain, Gwangyang City, Jeollanam-do, Korea, in September 2008. A voucher specimen (SNU-0785) has been deposited in the Herbarium of the Medicinal Plant Garden, College of Pharmacy, Seoul National University.

The air-dried barks (4.5 kg) were extracted with 80% methanol (MeOH) by ultrasonication at room temperature, and the methanolic extract was concentrated in vacuo to yield a crude extract (329.3 g). The methanolic extract was suspended in water and successively partitioned with *n*-hexane, ethyl acetate (EtOAc), and *n*-butanol (*n*-BuOH). The EtOAc fraction (78.2 g) was subjected to column chromatography on a silica gel column using mixtures of chloroform (CHCl_3_)-MeOH of increasing polarity as eluents to yield 9 subfractions (E1~E9). E3 was applied on a silica gel column eluted with (CHCl_3_-MeOH, 100:1 → 1:1) to yield 12 subfractions (E3–1~E3–12), and E3–6 afforded compound **1** (150 mg) on the C_18_ RP high performance liquid chromatography (HPLC) (MeOH-H_2_O, 95:5). The isolated compound **1** was identified as KWV by comparing the spectral data obtained from proton (^1^H)- and ^13^C-nuclear magnetic resonance (^13^C-NMR), and mass spectrometry (MS) with those reported in the literature [31]. Chemicals listed above are purchased from Sigma-Aldrich, MO, USA.

### KWV preparation

C_40_H_38_O_8,_ Yellowish amorphous powder. ESI MS (*m/z*): 645 [M—H]^-^, 647 [M + H]^+^. ^1^H-NMR (500 MHz, acetone-*d*_*6*_): δ 14.12 (1H,-OH), 12.78 (1H,-OH), 7.87 (1H, d, *J* = 9.0 Hz, H-6′), 7.83 (1H, d, *J* = 8.95 Hz, H-14″), 7.71 (1H, d, *J* = 15.3 Hz, H-β), 7.64 (2H, d, *J* = 8.4 Hz, H-2,6), 7.63 (1H, d, *J* = 15.5 Hz, H-α), 7.13 (2H, d, *J* = 8.5 Hz, H-16″,20″), 6.85 (2H, d, *J* = 8.6 Hz, H-3,5), 6.68 (2H, d, *J* = 8.5 Hz, H-17″,19″), 6.44 (1H, d, *J* = 8.9 Hz, H-13″), 6.31 (1H, d, *J* = 8.9 Hz, H-5′), 5.57 (1H, br s, H-3″), 5.08 (1H, m, H-22″), 4.4 (1H, dd, *J* = 6.6, 6.95 Hz, H-4″), 4.36 (1H, br s, H-3″), 3.69 (1H, br d, *J* = 6.0 Hz, H-5″), 3.13 (2H, d, *J* = 6.85 Hz, H-21″), 2.42 (1H, dd, *J* = 5.4, 17.9 Hz, H-6″), 2.22 (1H, dd, *J* = 6.1, 17.9 Hz, H-6″), 1.82 (3H, s, H-7″), 1.63 (3H, s, H-25′), 1.53 (3H, s, H-24″). ^13^C-NMR (125 MHz, acetone-*d*_*6*_): δ 208.0 (C-8″), 193.3 (C = O), 166.5 (C-2′), 164.7 (C-4′), 164.4 (C-10″), 163.2 (C-12″), 161.4 (C-4), 157.0 (C-18″), 145.4 (C-β), 137.2 (C-15″), 135.7 (C-1″), 132.2 (C-2), 132.2 (C-6), 131.8 (C-23″), 131.6 (C-6′), 131.2 (C-14″), 129.6 (C-16″), 129.6 (C-20″), 128.0 (C-1), 123.7 (C-22″), 123.5 (C-2′), 118.9 (C-α), 117.2 (C-3), 117.2 (C-5), 116.4 (C-17″), 116.4 (C-19″), 116.2 (C-11″), 116.0 (C-3′), 114.7 (C-9″), 114.4 (C-1′), 109.9 (C-5′), 108.6 (C-13″), 50.4 (C-4″), 40.8 (C-5″), 36.8 (C-6″), 33.9 (C-3″), 26.2 (C-25″), 24.2 (C-7″), 22.6 (C-21″), 18.3 (C-24″).

KWV solution was prepared in MeOH at a concentration of 0.02 mg/mL and was filtered through 0.45 μm membrane filter (Nylon, 170 mm; Millipore, MA, USA) before analysis with a Finnigan Surveyor HPLC-diode array detection (DAD) system that consisted of a pump, an autosampler, a PDA plus detector, and a Finnigan LCQ advantage MAX with Xcalibur software. A Shiseido CapCell PAK C18 column (5 μm, 4.6 mm i.d. ×150 mm) was used for chromatographic separation, with the column temperature set at 30°C. The mobile phase consisted of (A) Millipore-purified water and (B) HPLC-grade solvent acetonitrile (Fisher Scientific, Seoul, Korea) at a flow rate of 0.3 mL/min, with gradient elution as follows: 0 min, 20% B; 30 min, 90% B; 31 min, 20% B; 35 min, 20% B; v/v) for purity of compounds. In result, the purity of KWV was 96% by normalization of the peak areas detected by HPLC–DAD analysis.

### Neural stem cell culture

NSCs from the cortex of Sprague–Dawley rat (Orient Bio Inc., Gyeonggi-do, Korea) embryos at embryonic day 14 (E14) were isolated and cultured as previously described [36]. Animal experiments were performed in accordance with Chung-Ang University and NIH standards of animal care and approved by Chung-Ang University animal care and use committee (Permit Number: 13–0049, 2014–00032). The isolated NSCs (200,000 cells/mL) formed into neurospheres over 7 days in Dulbecco’s modified Eagle’s medium (DMEM)/F12 media supplemented with 2% (v/v) B27 (Gibco, NY, USA), 20 ng/mL EGF (Chemicon, CA, USA) and 20 ng/mL FGF2 (Chemicon). NSCs were propagated as neurospheres with media replacement every 2 days and passaging every 7 days. To induce differentiation, neurospheres were dissociated into a single-cell suspension with accutase (Chemicon) for 10 min at 37°C and plated onto 0.01% poly-d-lysine (PDL; Sigma-Aldrich) and 10 μg/mL laminin (Invitrogen, CA, USA) with no growth factors but supplemented with 2% B27 for 4 days at 37°C in 5% CO_2_. Cells were treated with KWV or vehicle (0.1% dimethyl sulfoxide [DMSO]; Sigma-Aldrich).

### Immunocytochemistry and cell counting

Immunocytochemical examination was performed as previously described [37]. Cell cultures were fixed with 4% paraformaldehyde (PFA; USB Products, OH, USA) for 30 min and washed with phosphate-buffered saline (PBS). Fixed cells were blocked with 5% normal goat serum (Millipore, CA, USA) and 0.2% Triton X-100 (Amresco, OH, USA) in PBS for 30 min and incubated with antibodies against βIII Tubulin (TuJ1, mouse monoclonal antibody, 1:1000; Sigma-Aldrich), glial fibrillary acidic protein (GFAP, rabbit polyclonal antibody, 1:1000; Dako, Copenhagen, Denmark), S100β (mouse monoclonal antibody, 1:500; Sigma-Aldrich), and Ki67 (rabbit monoclonal antibody, 1:400; Thermo Scientific, CA, USA) for 1 h. After PBS rinses, the cells were incubated for 30 min with secondary antibodies conjugated to Alexa Fluor 488 (goat anti-mouse immunoglobulin G [IgG], 1:1000; Invitrogen), Alexa Fluor 546 (goat anti-mouse IgG_1_, 1:1000; Invitrogen) or Cy3 (goat anti-rabbit IgG, 1:1000; Jackson ImmunoResearch, PA, USA) followed by 5 min in 4′,6-diamidino-2-phenylindole (DAPI, 1:10000 in PBS; Sigma-Aldrich) to stain the nuclei. Images were obtained using an inverse fluorescence microscope (DMIL; Leica, Hesse, Germany). TuJ1-, GFAP-, S100β-, or Ki67-positive cells were counted and normalized to total DAPI-positive cell numbers.

### Reverse-transcription polymerase chain reaction

The total RNA was extracted using TRIzol reagent (Invitrogen). First-strand cDNA was synthesized from 1 μg of total RNA with QuantiTect Reverse Transcription (RT) Kit (Qiagen, Limburg, Netherlands). Real-time polymerase chain reaction (PCR) was performed using the iQ SYBR Green supermix (Bio-Rad, CA, USA). The PCR conditions were as follows: initial activation at 95°C for 3 min followed by 40 cycles of denaturation at 95°C for 10 s, annealing at 58°C for 15 s, and extension at 72°C for 20 s. The primer sets used to amplify cDNA are described in the Supporting Information (S1 Table). The housekeeping gene *gapdh* was used as the internal control. The ratio of gene expression between NSCs treated with DMSO and those treated with KWV was calculated using the following formula: ratio = 2^ΔC(t) DMSO/ΔC(t) KWV^. Here, ΔC(t) DMSO = C(t) target gene—C(t) *gapdh*, from DMSO-treated NSCs, and ΔC(t) KWV = C(t) target gene—C(t) *gapdh*, from KWV-treated NSCs (C[t], threshold cycle).

### Western blot analysis

The NSCs were rinsed with PBS and lysed in a buffer (50 mM HEPES, 5 mM EDTA, 50 mM NaCl, 1% Triton X-100, and 1% NP-40 [all from Amresco]) in the presence of phosphatase inhibitors, 1 mM phenylmethylsulfonyl fluoride, 0.01 mg/mL leupeptin, and 0.01 mg/mL aprotinin (all from Sigma-Aldrich). Cell lysates were boiled for 5 min in sodium dodecyl sulfate (SDS) sample buffer (60 mM Tris-HCl of pH 6.8, 25% glycerol, 2% SDS, 0.1% bromophenol blue [all from Amresco], and 14.4 mM β-mercaptoethanol [Bio-Rad]) and separated by SDS-polyacrylamide gel electrophoresis. The proteins were electrophoretically transferred to polyvinylidene difluoride membranes (Millipore, MA, USA) for 2 h, which were then blocked in 5% nonfat dry milk or bovine serum albumin (Millipore, IL, USA) in PBS containing 0.05–0.1% Tween 20 (Amresco) for 30 min followed by overnight incubation at 4°C with antibodies against βIII Tubulin (TuJ1, 1:2000), Ki67 (1:200), phospho-ERK1/2 (rabbit monoclonal antibody, 1:1000; Cell Signaling, MA, USA), and GAPDH (mouse monoclonal antibody, 1:1000; Santa Cruz, CA, USA) and with horseradish peroxidase-conjugated goat anti-mouse or anti-rabbit IgG secondary antibodies (1:5000; Santa Cruz) for 1 h at room temperature. Protein bands were visualized on X-ray film using Western Blotting Luminol Reagent (Santa Cruz).

### Cell viability assay

Cell viability was assessed using the thiazolyl blue tetrazolium bromide (MTT; Sigma-Aldrich) assay. After neurosphere expansion for 1 week, the NSCs were dissociated and 3 × 10^4^ cells were plated onto PDL/laminin-coated 48-well plates (Corning, NY, USA) for 24 h followed by DMSO or KWV treatment for 48 h. The MTT solution (1 mg/mL) was added to each well for 2 h incubation at 37°C. Formazan crystals formed in NSCs were solubilized with 20% SDS in 50% aqueous *N*,*N*-dimethylformamide (Sigma-Aldrich). Lysates were transferred to 96-well plates (Corning) and absorbance was measured at 550 nm using the Synergy H1 Hybrid Multi-Mode Microplate Reader (Biotek, VT, USA).

### Proliferation assay

One day after plating, NSCs were treated with DMSO or KWV (0.5 μM) for 48 h and 5-bromo-2-deoxyuridine (10.0 μM, BrdU; Sigma-Aldrich) was added to the media either during 0–12 h or during 12–24 h. After an additional 2 days of differentiation, the cells were fixed with 4% PFA for 30 min and then fixed with 70% ice-cold MeOH (Merck, Hesse, Germany) for 10 min at room temperature. The cells were exposed to 2 M HCl (Sigma-Aldrich) for 20 min at 37°C and then incubated with 0.1 M sodium borate (Sigma-Aldrich) for 10 min at room temperature. Cells were washed with PBS containing 0.2% Triton X-100 and incubated with 5% normal goat serum and 0.2% Triton X-100 in PBS for 1 h, followed by overnight incubation at 4°C with an anti-BrdU antibody (mouse monoclonal antibody, 1:2000; Sigma-Aldrich). After rinsing, the cells were incubated with Alexa Fluor 488-conjugated secondary antibody for 30 min followed by DAPI for 5 min. The images were acquired with an inverse fluorescence microscope. BrdU-positive cells were counted and normalized to total cell number.

### Cell cycle analysis with fluorescence-activated cell sorting

After 2 days of treatment with DMSO or KWV, NSCs were dissociated to single cells using accutase and centrifuged (200 × *g*, 5 min, 4°C). The collected NSCs were resuspended to single cells in PBS and fixed with 70% ice-cold ethanol (Merck) for 1 h at 4°C. The NSCs were centrifuged and resuspended in PBS containing 0.05% Triton X-100, RNaseA (10 μg/mL) and propidium iodide (50 μg/mL) (both from Sigma-Aldrich) for 10 min. NSCs were collected by centrifugation, washed twice with PBS, and resuspended in PBS at 20,000 cells/500 μL for fluorescence-activated cell sorting (FACS) analysis (FACSCalibur; Becton Dickinson, CA, USA).

### Reactive oxygen species quantification

After one week of neurosphere expansion, NSCs were dissociated and plated onto PDL/laminin-coated 48-well plates (1 × 10^4^ cells/well) and treated with DMSO or KWV for 24 h in the presence of EGF and FGF2. Dichlorofluorescein-diacetate (10.0 μM, DCF-DA; Sigma-Aldrich) was added to each well for 30 min at 37°C followed by washing. Detection of fluorescence was performed using the inverse fluorescence microscope.

### Neurosphere growth rate

Neurosphere growth was measured as previously described [37]. Briefly, individual spheres (approximately 100–200 μm in diameter) were transferred to a single well of 96-well plates containing 200 μL of growth media (n = 4, for each treatment). Neurospheres were treated with DMSO or KWV and the diameter of the spheres was measured daily using a lens-mounted microscope (Leica). The volume of each sphere was calculated as an index of cell number using the equation V = 4/3πr^3^, where r = 1/2 diameter, as described in detail [38].

### Neurosphere formation assay

Primary NSCs were dissociated and plated as single cells onto uncoated 48-well plates at a density of 10 cells/μL in 250 μL of media in each well (n = 6, for each treatment). The cells were grown as floating cell aggregates (neurospheres) for 7 days in growth media containing DMSO or KWV. The number of neurospheres was counted from 6 independent fields photographed using the inverted microscope (Leica) and JULI-Digital Bio (NanoEnTek, Seoul, Korea).

### Detection of microRNA expression

The miScript PCR system (Qiagen) was used to analyze the expression of miRNAs, including rno-miR-9, rno-miR-29a, rno-miR-124 and rno-miR-181a, according to the manufacturer’s instructions. The total RNA including miRNA was extracted using TRIzol reagent. cDNA was generated from 2 μg of total RNA using the miScript II RT kit. Real-time PCR was performed using the miScript SYBR Green PCR kit with 5 miScript Primer Assays of Rn_miR-9_1, Rn_miR-29a*_2, Rn_miR-124*_1, Rn_miR-181a_2, and Hs_RNU6–2_11. The PCR reaction was conducted at 95°C for 15 min, followed by 40 cycles of 94°C for 15 s, 55°C for 30 s, and 70°C for 30 s. U6 small nuclear RNA (RNU6) was used for normalization.

### Statistical analysis

Data were expressed as mean ± standard error of mean (SEM) or standard deviation (SD). Statistical significance was determined by Student’s *t*-test (*P < 0.05, **P < 0.01).

## Results

### KWV promotes neuronal differentiation in NSCs

**Fig 1:**
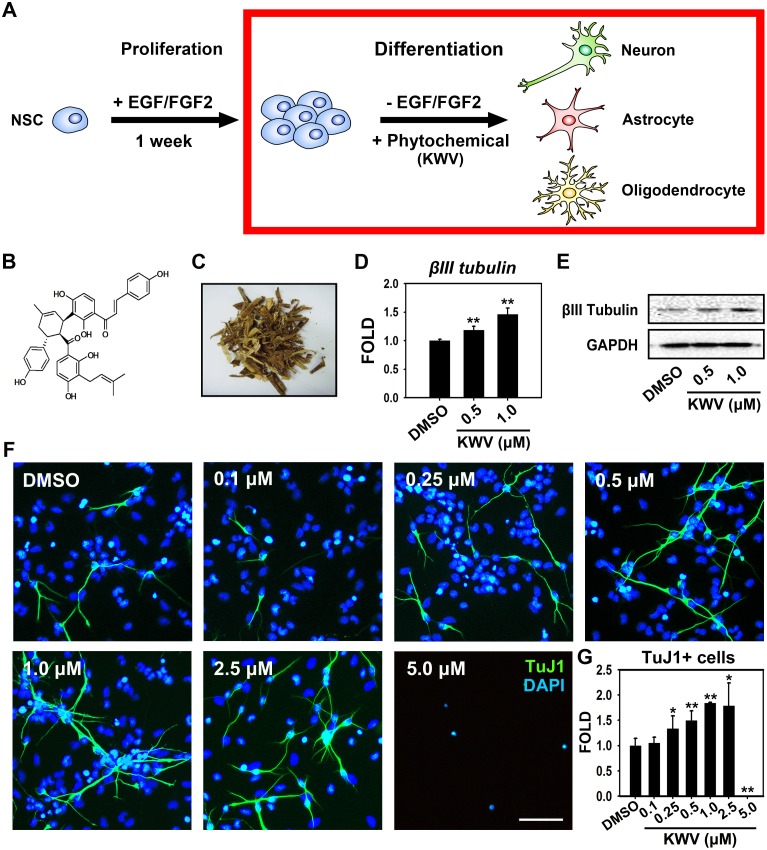
KWV increases neurogenesis during NSC differentiation. (A) Experimental scheme. NSCs were expanded as neurospheres in the presence of EGF and FGF2 for one week. To explore the roles of KWV during NSC differentiation, neurospheres were dissociated and plated as single cells and treated with vehicle (0.1% DMSO) or KWV and differentiated in the absence of growth factors. (B) The chemical structure of KWV. (C) A photo of air-dried stem barks of *M. bombycis*. (D) Bar graph shows the relative mRNA expression values of *βIII tubulin*. Total RNA was isolated from the cells treated with DMSO or KWV (0.5 or 1.0 μM) for 2 days of differentiation. cDNA was synthesized and subjected to real-time PCR using specific primers for *βIII tubulin*. *Gapdh* was used as an internal control. The data were expressed as mean ± SD (n = 3). (E) The representative band image for the protein levels of βIII Tubulin. Two days after treatment, total cell lysates from differentiated NSCs were subjected to western blot analysis with TuJ1 antibody. (F) Representative immunofluorescence images of NSCs differentiated for 4 days in the presence of DMSO or KWV (0.1–5.0 μM). Cells were immunostained with TuJ1 antibody and nuclei were identified by DAPI staining [TuJ1-positive neurons (green), nuclei (blue)]. Scale bar, 50 μm. (G) Quantification of neurons. TuJ1-positive cells were counted and normalized to total DAPI-positive cell numbers. KWV-treated NSC numbers were divided by DMSO-treated NSC numbers to yield fold changes. Values were presented as mean ± SEM (n = 3). Statistical analysis of all data was performed using the Student’s *t*-test (*P < 0.05 and **P < 0.01 vs. control).

NSCs derived from the developing rat cortex at E14 were maintained as free floating aggregates, termed neurospheres, which can be propagated for several months in the presence of growth factors such as EGF and FGF2 [39]. While NSCs spontaneously differentiate upon growth factor withdrawal, we screened several phytochemicals to find active natural compounds that could enhance this differentiation. Neurospheres were expanded for one week in EGF and FGF2 and then differentiated for 4 days in the absence of growth factors and in the presence of 14 separate phytochemicals at 0.5 μM [KWV, *ent*-16α*H*,17-hydroxy-kauran-19-oic acid, *ent*-16β,17-dihydroxy-kauran-19-oic acid, kirenol, quercetin-3,4′,7-O-trimethyl ether, hinokiflavone, idescarpine, idesolide, dehydrohirsutanonol, oregonin, 1,7-bis(3,4-dihydroxyphenyl)-5-hydroxyheptan-3-one, curcumin, lup-20(29)en-2,28-diol-3-yl caffeate, and 2′-O-acetylsalicortin] (Fig. 1A).

Of the tested compounds, KWV, a chalcone-derivative phenolic compound (Fig. 1B) isolated from the *M. bombycis* bark (Fig. 1C) appeared to have a neurogenic effect (Fig. 1D-1G). Quantifying the mRNA expression levels of the neuronal gene *βIII tubulin* by RT PCR revealed that NSCs treated with 0.5 or 1.0 μM KWV showed a 1.2- or 1.5-fold increase, respectively, compared to DMSO vehicle-treated controls (Fig. 1D). Protein level assessment by western blot analysis showed that cells treated with 0.5 or 1.0 μM of KWV during differentiation also had increased levels of the neuronal protein βIII Tubulin compared to DMSO-treated controls (Fig. 1E).

KWV at both 0.5 and 1.0 μM significantly affected neuronal differentiation compared to the DMSO control and, though not significant, the effect appeared greater with the higher KWV concentration. To assess whether KWV increases neuronal differentiation in a dose-dependent manner and to extrapolate the optimal KWV concentration, cells were treated with KWV at 0.1, 0.25, 0.5, 1.0, 2.5 or 5.0 μM. Immunostaining and quantification of TuJ1-positive cells demonstrated that neuronal differentiation was significantly greater with KWV treatment at 0.25, 0.5, 1.0 and 2.5 μM compared to DMSO treatment (Fig. 1F and 1G). In contrast, the neuron number was not significantly different from DMSO control at 0.1 μM and, importantly, no neurons were generated at 5.0 μM presumably due to KWV toxicity at this higher dose (Fig. 1F and 1G).

**Fig 2:**
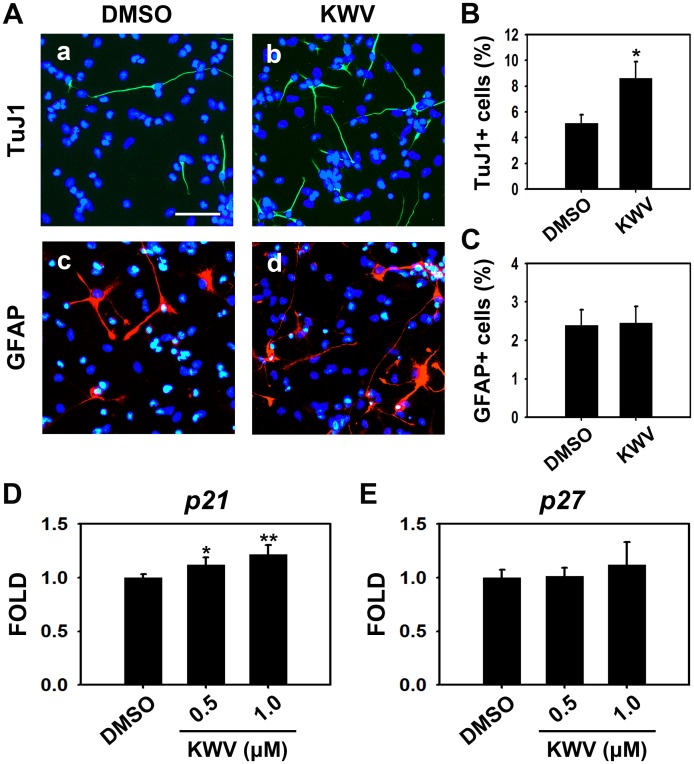
KWV enhances neurogenesis but not astrocytogenesis during NSC differentiation. (A) Representative immunofluorescence images of NSCs differentiated for 4 days in the presence of DMSO or 0.5 μM KWV [TuJ1-positive neurons (a and b, green), GFAP-positive astrocytes (c and d, red), nuclei (blue)]. Scale bar, 50 μm. (B, C) Quantification of neurons or astrocytes. TuJ1 positive or GFAP expressing cells were counted and normalized to total cell number. All cell count data were expressed as mean ± SD (n = 3). Bar graphs show the relative mRNA expression levels of *p21* (D) and *p27* (E). *Gapdh* was used as an internal control. Values were mean ± SD (n = 4). Statistical analysis of all data was performed using the Student’s *t*-test (*P < 0.05 and **P < 0.01 vs. control).

NSCs have the capacity to differentiate into neurons and glia. Clearly KWV increases the differentiation propensity of NSCs into neurons in a dose-dependent manner. To determine if KWV affected other neural cell types, we differentiated NSCs in the presence of 0.5 μM KWV and examined both neuronal specific protein, βIII Tubulin (detected by TuJ1 antibody) and astrocyte-specific protein, GFAP. Immunostaining showed that KWV treatment significantly increased the number of TuJ1-positive neurons without affecting the number of GFAP-expressing astrocytes compared to DMSO treatment (Fig. 2A-2C). Immunocytochemistry results obtained by detecting another astrocyte marker S100β showed that both numbers and morphologies of S100β-positive cells in KWV treated condition were not significantly different from those of control (S1A and S1B Fig.). RT PCR data using *s100β* specific primers confirmed that KWV treatment did not alter the propensity of astrocyte differentiation in NSCs (S1C and S1D Fig.). Collectively, these data suggest that KWV at doses below 5.0 μM is not toxic to NSCs and that KWV treatment increases neuronal differentiation, while not affecting glial differentiation, in the absence of growth factors.

Expression levels of *p21* determined by RT PCR significantly increased whereas those of *p27* remained similar when NSCs were differentiated in the presence of 0.5 or 1.0 μM of KWV (Fig. 2D and 2E). When NSCs were differentiated in the presence of 1.0 μM KWV for 2 days and analyzed by FACS, KWV increased the percentage of G_0_-G_1_ phase cells and decreased G_2_-M phase cells compared to DMSO-treated control (S2 Fig.).

### KWV increases cell survival during NSC differentiation

**Fig 3:**
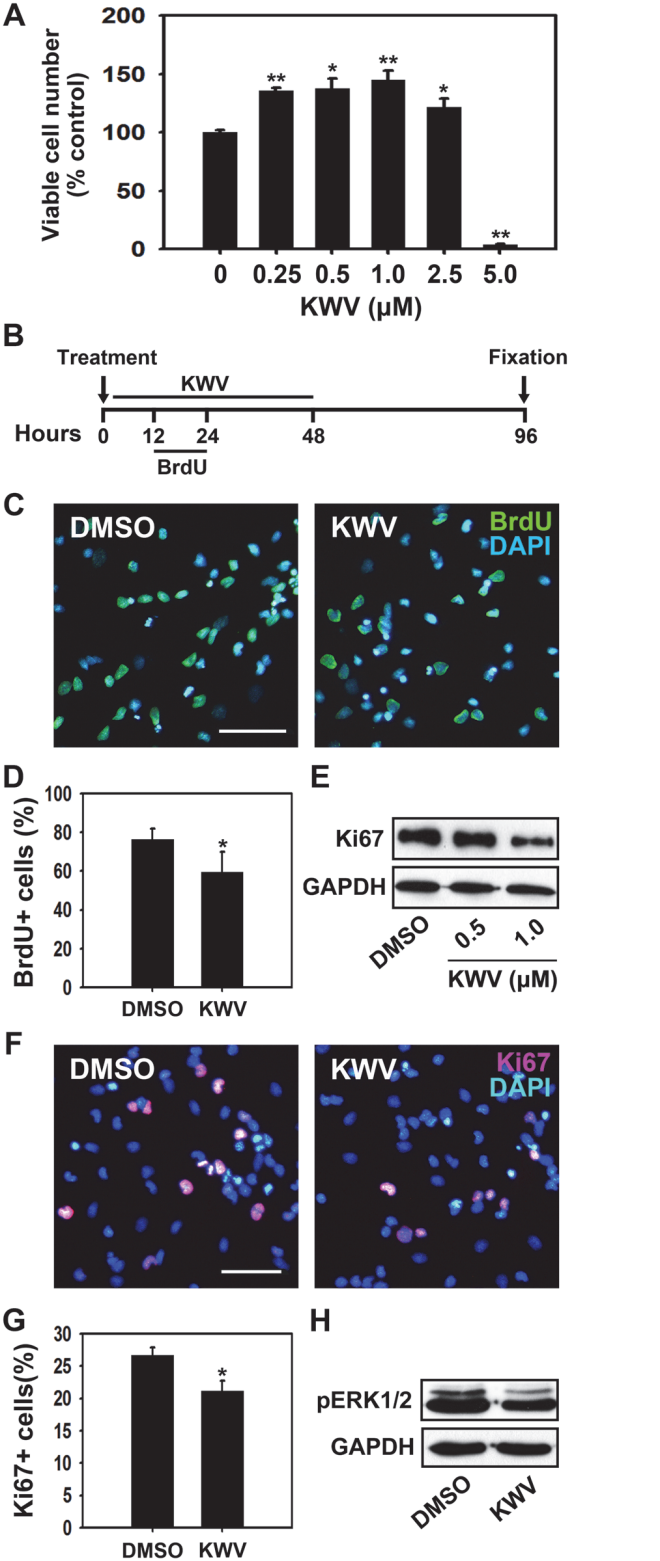
KWV promotes cell survival rather than proliferation during NSC differentiation. (A) Cell viability was assessed using the MTT assay after 2 days of NSC differentiation in the presence of DMSO or various concentrations of KWV (0.25–5.0 μM). The values were presented as mean ± SD (n = 3). (B) NSCs were treated with DMSO or KWV (0.5 μM) for 2 days and BrdU (10.0 μM) was added to the media between 12–24 h during the differentiation. After an additional 2 days of differentiation, cells were fixed and immunostained with anti-BrdU antibody. (C) Representative immunofluorescence images of BrdU-positive cells (green) and nuclei (blue). Scale bar, 50 μm. (D) Quantification of BrdU-positive cells. BrdU-positive cells were counted and normalized to total cell number. Cell count data were expressed as mean ± SD (n = 3). (E) Western blot analysis of Ki67 in NSCs treated with DMSO or KWV (0.5 or 1.0 μM) for 8 h of differentiation. (F) Representative immunofluorescence images of Ki67-positive cells (red) and nuclei (blue). NSCs differentiated for 2 days in the presence of DMSO or 0.5 μM KWV were immunostained with anti-Ki67 antibody. Scale bar, 50 μm. (G) Quantification of Ki67-positive cells. Ki67-positive cells were counted and normalized to total cell number. Data were presented as mean ± SD (n = 3). (H) Western blot analysis of phosphorylated ERK1/2 in NSCs treated with DMSO or 0.5 μM KWV for 4 h of differentiation. Statistical analysis of all data was performed using the Student’s *t*-test (*P < 0.05 and **P < 0.01 vs. control).

For KWV-induced increased neuronal differentiation to be of benefit to neurodegenerative diseases, it requires that KWV does not reduce overall cell viability. To address this concern, a MTT assay was used to quantify the viability of NSCs differentiated and treated with KWV at 0.25, 0.5, 1.0, 2.5 or 5.0 μM in the absence of growth factors. Results showed that KWV treatment at 0.25, 0.5, 1.0 or 2.5 μM did not reduce but rather increased cell viability compared to DMSO-treated control cells and confirmed that KWV at 5.0 μM was toxic to NSCs (Fig. 3A).

A rise in cell numbers could be due to facilitated proliferation or to increased cell protection leading to reduced cell death. Pulsing with BrdU, which is a synthetic nucleoside analogous to thymidine that incorporates into newly synthesized DNA upon cell replication, was performed to determine whether increased proliferation during differentiation underlay the increased cell viability. When BrdU was added during the first 12 h of 0.5 μM KWV treatment, the number of BrdU-positive cells was not significantly different from that of DMSO-treated control (S3A-S3C Fig.), suggesting no effect on NSC proliferation at the early time point of differentiation. Interestingly, we observed that KWV enhanced both BrdU-positive and TuJ1-positive cells when BrdU was added for the first 12 h during differentiation (S3D and S3E Fig.). KWV did not change the numbers of BrdU-positive and GFAP-expressing cells significantly from DMSO treated control (S3F and S3G Fig.). In contrast, when BrdU was added during the next 12 h of KWV treatment (Fig. 3B), the number of BrdU-positive cells was significantly less than that of the control (Fig. 3C and 3D), showing that KWV actually inhibits NSC proliferation during differentiation. These data suggest that KWV increases neurogenesis and the final cell division mainly occurs within 12 h of KWV treatment. A commonly used marker of cell proliferation is Ki67, a nuclear protein detected during all the phases of cell cycle (G_1_, G_2_, and S phases and mitosis) except the resting state (G_0_). Western blot analysis of differentiating NSCs treated with 0.5 or 1.0 μM of KWV for 8 h showed that the production of Ki67 protein was reduced in a dose-dependent manner (Fig. 3E). Subsequent quantification of Ki67 immunostained cells showed that treatment of KWV (0.5 μM) for 2 days significantly reduced the number of Ki67-positive cells compared to that of control, confirming the anti-proliferative effect of KWV during differentiation (Fig. 3F and 3G). Finally, ERK1/2 phosphorylation by mitogens is generally known to promote proliferation and inhibit neuronal differentiation of NSCs [14, 40]. Western blot analysis showed decreased phosphorylation of ERK1/2 in 4 h of 0.5 μM KWV-treated cells (Fig. 3H), again suggesting that KWV reduces cell proliferation during differentiation. Collectively, these results showed that NSCs treated with KWV in the absence of EGF and FGF2 have increased viability yet reduced proliferation, suggesting that KWV enhances protection but not proliferation of NSCs during differentiation.

### KWV inhibits NSC proliferation in the presence of EGF and FGF2

**Fig 4:**
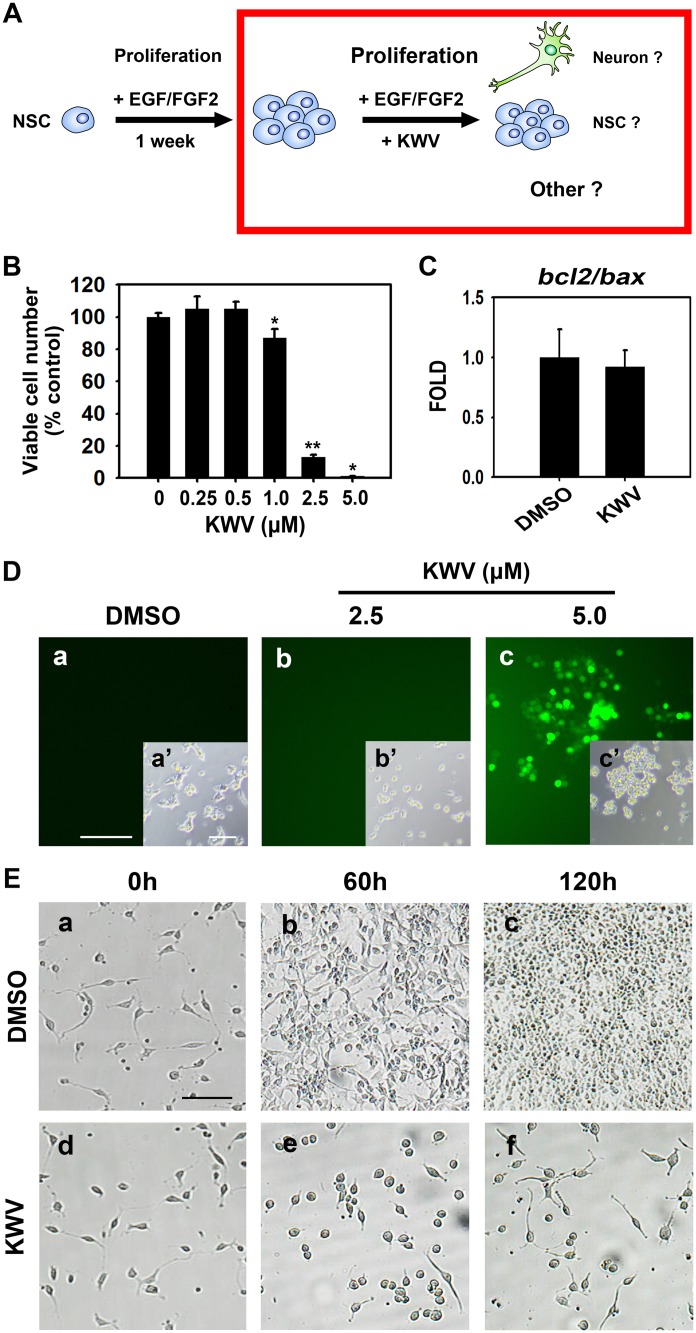
KWV reduces cell number during NSC proliferation. (A) After 1 week of expansion, NSCs were dissociated, plated and treated with DMSO or KWV in the presence of EGF and FGF2 to determine the effects of KWV during NSC proliferation. (B) Cell viability was assessed using the MTT assay after 2 days of DMSO or KWV (0.25–5.0 μM) treatment in the presence of growth factors. Values were mean ± SD (n = 3). (C) The mRNA ratio of *bcl2* to *bax* in proliferating NSCs treated with DMSO or 2.5 μM KWV for 2 days. *Gapdh* was used as an internal control. The data were expressed as mean ± SD (n = 4). (D) Representative fluorescence images (a-c) of DCF-DA added proliferating NSCs in the presence of DMSO or KWV (2.5 or 5.0 μM). Insets are phase contrast images (a’-c’). (E) Representative digital images of NSCs treated with DMSO or 2.5 μM KWV in the presence of EGF and FGF2 at 0, 60, and 120 h. The scale bars represent 50 μm in (D) and (E). Statistical analysis of all data was performed using the Student’s *t*-test (*P < 0.05 and **P < 0.01 vs. control).

NSCs typically reduce proliferation when undergoing cellular differentiation and KWV treatment clearly enhanced both in the absence of mitogens. As the presence of mitogens generally maintains NSCs in a proliferative state, we next wanted to determine whether KWV affected NSC proliferation even in the presence of EGF and FGF2 (Fig. 4A). First, a MTT assay was used to assess whether KWV treatment affected cell viability during proliferative conditions. In contrast to the increased viability observed during differentiation without mitogens (Fig. 3A), NSCs treated with 0.25 and 0.5 μM of KWV did not show increased viability in the presence of EGF and FGF2 (Fig. 4B). Interestingly, the viability of NSCs markedly decreased to 13.1% of control when NSCs were treated with 2.5 μM of KWV (Fig. 4B). To examine whether this decrease of cell viability was due to toxicity from 2.5 μM KWV, real-time PCR was performed to measure the mRNA expression levels of the pro-apoptotic gene, *bax*, and the anti-apoptotic gene, *bcl2* (Fig. 4C). In addition, DCF-DA staining was used to measure reactive oxygen species (ROS) (Fig. 4D). Surprisingly, although NSCs treated with 2.5 μM KWV in the presence of growth factors showed poor viability, there was no significant difference in the expression of either gene or in ROS production compared to DMSO-treated cells (Fig. 4C and 4D). In contrast, NSCs treated with 5.0 μM of KWV showed poor viability and concomitantly produced high levels of ROS (Fig. 4B and 4D), suggesting that 5.0 μM KWV is toxic to NSCs not only in the absence but also in the presence of mitogens. Observation of NSC morphology following 2.5 μM KWV treatment in the presence of mitogens showed a clear reduction in the number of cells but the cells appeared to be healthy (Fig. 4E). This suggests that while 2.5 μM of KWV is not toxic to NSCs, it may inhibit NSC proliferation and enhance NSC differentiation even in the presence of EGF and FGF2.

**Fig 5:**
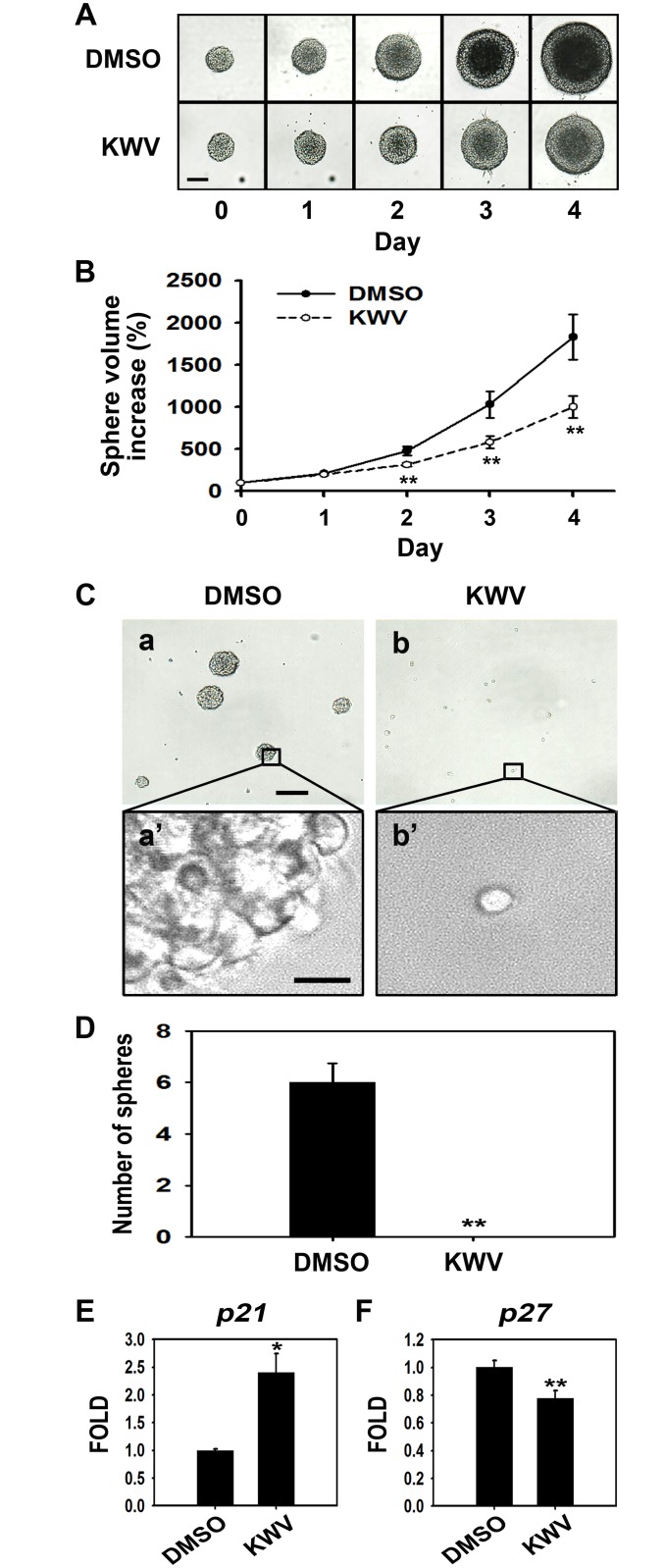
KWV inhibits NSC proliferation in the presence of EGF and FGF2. (A) Digital images of neurospheres treated with either DMSO or 2.5 μM of KWV for 5 days in the presence of EGF and FGF2. Scale bar, 100 μm. (B) The volume of neurospheres was calculated by measuring the diameter of individual neurospheres treated with DMSO (black circle) or KWV (2.5 μM, white circle). Values were presented as mean ± SD (n = 4, for each treatment). (C) Neurosphere formation assay. Primary NSCs were dissociated and plated as single cells at clonal density (10 cells/μL). The NSCs were grown for 7 days in the presence of mitogens added with DMSO or 2.5 μM KWV. The boxed areas in (a) and (b) are magnified in (a’) and (b’), respectively. The scale bars represent 100 μm in (a) and (b) and 10 μm in (a’) and (b’). (D) Bar graph depicts the number of neurospheres counted from 6 independent randomly chosen fields. Cell count data were expressed as mean ± SD (n = 6, for each treatment). Bar graphs show the relative mRNA expression levels of *p21* (E) and *p27* (F) in NSCs treated with DMSO or 2.5 μM KWV for 2 days in the presence of growth factors. *Gapdh* was used as an internal control. The values were mean ± SEM (n = 3). Statistical analysis of all data was performed using the Student’s *t*-test (*P < 0.05 and **P < 0.01 vs. control).

NSCs in the presence of EGF and FGF2 are maintained as neurospheres, whose diameter can be measured to calculate neurosphere volume. In order to determine whether KWV inhibited proliferation even in the presence of mitogens, we measured the sizes of neurospheres treated with DMSO or 2.5 μM of KWV starting on the day of passage (day 0) and for 4 consecutive days (Fig. 5A and 5B). On day 0 and 1, neurospheres treated with DMSO or KWV had the similar volume. By day 2 of growth and KWV treatment, however, the volume of the KWV-treated NSCs was significantly less than that of the DMSO-treated control (Fig. 5A and 5B). Furthermore, when NSCs were dissociated and plated in the presence of growth factors at clonal density (10 cells/μL) for 7 days, no observable neurospheres were generated from 2.5 μM of KWV-treated cells (Fig. 5C and 5D), suggesting a dramatic loss of cell proliferation. This was in stark contrast to DMSO-treated control cells that reformed several neurospheres (low magnification) displaying a healthy morphology (high magnification) (Fig. 5C and 5D). Finally, 2.5 μM of KWV significantly up-regulated the mRNA expression levels of *p21* in the presence of EGF and FGF2 (2.4-fold, vs. control, Fig. 5E). However, KWV decreased the expression levels of *p27* (0.8-fold, vs. control, Fig. 5F). Taken together, these data suggest that KWV at 2.5 μM inhibits the proliferation of NSCs and induces p21 even in the presence of mitogens.

### KWV induces neurogenesis in the presence of EGF and FGF2

**Fig 6:**
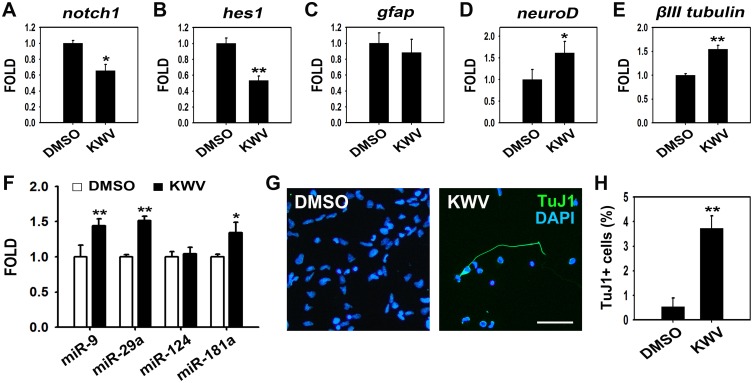
KWV induces neuronal differentiation in the presence of EGF and FGF2. After expansion for 1 week in the presence of EGF and FGF2, NSCs were dissociated and plated. Then cells were treated with either DMSO or KWV (2.5 μM) in the presence of growth factors for 2 days and the mRNA expression levels of *notch1* (A) and *hes1* (B) were shown by RT PCR followed by real-time PCR. *Gapdh* was used as an internal control. Data were presented as mean ± SEM (n = 3). The *gfap* (C), *neuroD* (D), and *βIII tubulin* (E) mRNA expression levels of NSCs treated with DMSO or 2.5 μM KWV for 24 h in the presence of mitogens. Values were expressed as mean ± SD (C and D; n = 4) or mean ± SEM (E; n = 3). (F) The expression of *miR-9*, *miR-29a*, *miR-124*, and *miR-181a* in cells treated with DMSO (white bar) or 2.5 μM KWV (black bar) for 24 h in the presence of growth factors. *RNU6* was used as a loading control. The values were mean ± SD (n = 4). (G) Representative immunofluorescence images of cells treated with DMSO or 2.5 μM KWV for 43 h in the presence of EGF and FGF2. Cells were immunostained with TuJ1 antibody and nuclei were identified by DAPI staining [TuJ1-positive neurons (green), nuclei (blue)]. Scale bar, 50 μm. (H) Quantification of neurons. TuJ1-positive cells were counted and normalized to total cell number. Cell count data were presented as mean ± SD (n = 3). Statistical analysis of all data was performed using the Student’s *t*-test (*P < 0.05 and **P < 0.01 vs. control).

As KWV clearly reduced proliferation even in the presence of mitogens, we next wanted to determine whether this reduced proliferation also led to increased cell differentiation. After treating NSCs with 2.5 μM KWV in the presence of EGF and FGF2, real-time PCR data showed that the genes *notch1* and *hes1*, which are expressed during NSC maintenance, were both significantly down-regulated compared to DMSO-treated controls (Fig. 6A and 6B). As with the differentiation results in the absence of mitogens, KWV treatment in the presence of mitogens did not lead to changes in *gfap* expression (Fig. 6C). In contrast, the genes *neuroD* and *βIII tubulin*, which are expressed during neuronal differentiation, were both significantly up-regulated compared to DMSO-treated controls (Fig. 6D and 6E). miRNAs are noncoding transcripts of small nucleotides that have been reported to regulate cell fate [41]. Assessment of the miRNA expression levels in 2.5 μM of KWV-treated NSCs in the presence of mitogens using RT PCR showed that *miR-9*, *miR-29a*, and *miR-181a* expression were significantly up-regulated (1.44-, 1.51- and 1.34-fold, vs. control, respectively), while *miR-124* expression was unchanged (Fig. 6F). Finally, immunostaining confirmed the PCR results, showing that NSCs treated with 2.5 μM KWV produced significantly more TuJ1-positive neurons even in the presence of EGF and FGF2 (Fig. 6G and 6H). In addition, the representative images of dissociated and plated NSCs clearly confirmed the reduced cell proliferation following KWV treatment that was reported within the NSC neurospheres (Figs. 5A-5D and 6G). Taken together, the data suggest that KWV is a potent natural reagent that enhances neuronal differentiation in the absence and, unexpectedly, in the presence of growth factors by overcoming the proliferation signals provided by mitogens.

## Discussion

The treatment or prevention of neurodegenerative diseases may be facilitated by endogenous neurogenesis from NSCs or by protection from neuronal death [21, 42]. Adult NSCs are found in the SVZ and the subgranular zone of the hippocampal dentate gyrus [1, 19]. Recent studies suggest that intrinsic or extrinsic factors and some small molecules can regulate the differentiation of NSCs into neurons, astrocytes, or oligodendrocytes [7, 12]. For instance, we recently reported that oxadiazole derivatives can induce astrocytogenesis [43], and it is studied that an aminopropyl carbazole named P7C3 has neurogenic and neuroprotective effects when administered in the hippocampus of adult mice [44]. The small molecule KHS101 is known to increase neuronal differentiation by interacting with the intracellular protein, named Transforming acidic coiled-coil [45]. Interestingly, some antidepressants have been discovered to induce neurogenesis [46–51] and the anticonvulsant valproate is also known to enhance neurogenesis and reduce NSC death [52–55]. In addition, many natural products, including ginsenosides isolated from *Panax notoginseng*, galcinol from the fruit rind of *Garcinia indica*, and MeOH extracts of *Nelumbo nucifera* rhizome, are reported to increase neurogenesis [56–59]. While the exact mechanisms how these compounds increase adult neurogenesis are still unclear, the discovery of additional compounds and mechanisms could benefit the regenerative medicine field.

Several small molecules isolated from components of the root of *Morus* species, such as cyclomulberrin, neocyclomorusin, sanggenon I, morusin, KWU, KWE, moracin P, moracin O, and mulberrofuran Q, are reported to protect human neuronal cells derived from the human neuroblastoma SH-SY5Y cell line [30, 60]. In addition, moracenin D isolated from Mori cortex radicis protected SH-SY5Y cells cultured in high dopamine concentrations by inducing *nurr1* mRNA expression and reducing *α-synuclein* mRNA expression [61]. In order to identify additional novel molecules that can regulate NSC fate, we screened several natural compounds and found that KWV, a phenolic compound isolated from the bark of *M. bombycis*, significantly increased neurogenesis without affecting astrogliogenesis.

Following mitogen withdrawal to initiate NSC differentiation, KWV treatment further inhibited the proliferation of NSCs, promoted the survival of neurons and induced neurogenesis. Generally, phosphorylation of ERK1/2 by mitogens is known to enhance proliferation and inhibit neuronal differentiation of rat NSCs [14, 40]. We showed that KWV inhibited phosphorylation of ERK1/2, providing one potential mechanism of action whereby KWV blocks proliferation and induces differentiation of NSCs.

KWV showed differential effects on cell viability depending on the absence or presence of EGF and FGF2. KWV at 2.5 μM increased the viability of differentiated NSCs up to 120% in the absence of EGF and FGF2. However, in the presence of growth factors when NSCs proliferate and maintain their multipotency [1], the viability of NSCs treated with 2.5 μM KWV was less than 20% when compared to that of control. Even in the presence of mitogens, KWV increased neuronal differentiation resulting in reduction of proliferation, thus the viability of NSCs appeared to be reduced. It is reported that the proliferative effect of FGF2 on NPCs is inhibited by valproic acid, and instead neurogenesis is induced via the β-catenin-Ras-ERK-p21^Cip/WAF1^ pathway [54]. Similar to valproic acid, KWV (2.5 μM) in the presence of EGF and FGF2 decreased NSC proliferation, demonstrated by a significant reduction in neurosphere formation and growth along with down-regulation of genes involved in cell proliferation such as *notch1* and *hes1*. Also similar to valproic acid, KWV induced an unexpected increase in neuronal differentiation even in the presence of mitogens, demonstrated by an up-regulation of neurogenesis-related genes including *neuroD*, *βIII tubulin*, and *p21*. The findings that KWV had an inhibitory effect on HIF-1 and that factors inhibiting HIF-1 were negative regulators of Notch signaling [33, 62], suggest that KWV may inhibit NSC proliferation and enhance neurogenesis through Notch inhibition in NSCs.

Several miRNAs are known to be involved in neurogenesis and cell proliferation. miR-9 is highly expressed in the developing and adult vertebrate brain as a versatile regulator of neurogenesis [63]. Hes1, which is involved in NSC maintenance, is a target of miR-9 and miR-9 transcription is negatively regulated by Hes1 [64, 65]. A potential mechanism for KWV-induced neurogenesis is down-regulated expression of *notch1* and *hes1*, which, in turn, relieves the repression of miR-9 transcription. Indeed, we found that KWV treatment led to both a significant decrease in *hes1* and *notch1* transcript expression and an increase in *miR-9* expression in NSCs. A recent study showed that miR-181a suppressed the expression of Prox1, which functions as a Notch1 inhibitor, and that up-regulation of Notch1 induced astrocyte differentiation from NPCs [66]. However, miR-181a overexpression in human dermal fibroblasts resulted in the increase of senescence marker p53 and p16, and subsequent fibroblast senescence [67]. While our results with rat NSCs did not support KWV induction of astrocyte differentiation, KWV treatment did inhibit NSC proliferation, which may be related to the *miR-181a* increase seen in the NSCs. The most abundant and best studied miRNA in the brain is miR-124 [68]. It is known that miR-124 antagonizes the anti-neural Small c-terminal domain phosphatase 1 and induces neurogenesis in P19 cells [69]. However, during spinal cord development, neither inhibition nor overexpression of miR-124 significantly altered the acquisition of neuronal fate, suggesting that it is not acting as a primary determinant of neural differentiation [70]. Although KWV is a potent neurogenic factor, we did not observe significant increase of *miR-124* suggesting that miR-124 is not involved in KWV mediated neuronal differentiation. It is reported that reduced expression of miR-29a was found in patients and animal models of neurodegenerative diseases such as Alzheimer’s disease and Huntington’s disease [71, 72]. A recent study reiterated the role of miR-29 in neuronal survival by knocking down miR-29 in the mouse brain [73]. miR-29 knockdown resulted in massive neuronal death in the hippocampus and cerebellum. KWV treatment increased the expression of miR-29a, providing a possible mechanism of increased neuronal survival during NSC differentiation. Repressor element-1 silencing transcription factor (REST), also known as neuronal restrictive silencing factor, is well known transcriptional regulator that represses neuronal differentiation related genes in non-neuronal cells [74, 75]. Recently, it is reported that miR-29a knockin in mesenchymal stem cells (MSCs) significantly down-regulated the expression of REST and generated neurons from MSCs, suggesting that miR-29a is involved in neuronal differentiation [76]. Thus, up-regulation of miR-29a upon KWV treatment may also play critical roles in KWV-induced neurogenesis.

In the present study, we showed that KWV inhibited the proliferation of NSCs, protected cells during differentiation and increased neurogenesis. Interestingly, the decrease in proliferation and increase in neurogenesis induced by KWV treatment of NSCs occurred even in the presence of EGF and FGF2, which suggests that KWV is a neurogenic factor potent enough to overcome mitogen effects. However, to be further developed as a drug candidate that modulates NSCs, the structure of KWV has to be modified to reduce the KWV toxicity at 5.0 μM. In addition, studies are required to further clarify mechanisms underlying the effects of KWV on NSCs. Nonetheless, adding KWV to the repertoire of natural compounds that can affect neuronal survival and differentiation may ultimately benefit patients suffering from neurodegenerative diseases.

## Supporting Information

S1 Fig KWV does not change the propensity of NSC differentiation to astrocytes. NSCs were expanded for 1 week, dissociated and plated for additional 4 days in the absence of growth factors and in the presence of KWV to induce differentiation. After fixation, cells were immunostained with anti-S100β antibody. (A) Representative images of cells treated with DMSO or KWV (0.5 μM) [S100β-positive cells (red), nuclei (blue)]. Scale bar, 50 μm. (B) Quantification of S100β-positive cells. The S100β-positive cells were counted and divided by the total number of nuclei. Data are presented as mean ± SD (n = 3). NSCs were expanded for 1 week, dissociated and plated for additional 2 days in the absence of growth factors and in the presence of DMSO or KWV. Total mRNA was extracted and RT PCR was performed. (C) mRNA expression levels of *s100β* in either DMSO or KWV (0.5 or 1.0 μM) treated cells. (D) mRNA expression levels of *s100β* in the presence of mitogens for 8 days (after 1 week of expansion, NSCs were dissociated and re-plated in the presence of mitogen and KWV for 1 day). Data are presented as the mean ± SD (n = 3). Statistical analysis was performed using the Student’s *t*-test.
(TIF)

S2 Fig KWV promotes cells to become G_0_-G_1_ status. NSCs were treated with DMSO (A) and 1.0 μM KWV (B) for 2 days in the presence of EGF and FGF2 and analyzed using the FACS system to determine the cell cycle. Among the three independent results, we are showing one representative data.
(TIF)

S3 Fig KWV does not increase NSC proliferation but increases neurogenesis during the first 12 h of differentiation. (A) Cells were treated with KWV (0.5 μM) for 2 days in the absence of EGF and FGF2. BrdU (10.0 μM) was added to the media during the first 12 h. (B) Representative immunofluorescence images of BrdU-positive cells (green) and nuclei (blue). (C) Quantification of BrdU-positive cells. (D) Representative photos of BrdU-positive cells (red), TuJ1-positive cells (green) and nuclei (blue). (E) Quantification of BrdU-positive and TuJ1-positive cells in DMSO or KWV (0.5 μM) treated cells. (F) Representative immunofluorescence images of BrdU-positive cells (green), GFAP-positive cells (red) and nuclei (blue) in DMSO or KWV treated cells. (G) Quantification of BrdU-positive and GFAP-positive astrocytes in DMSO or KWV treated cells. Scale bars, 50 μm (B, D, F). All cell count data were expressed as mean ± SD (n = 3). Statistical analysis was performed using the Student’s *t*-test (*P < 0.05).
(TIF)

S1 Table Real-time PCR primers. (DOCX)
