# Supplementary figures and images for: Kuwanon V Inhibits Proliferation, Promotes Cell Survival and Increases Neurogenesis of Neural Stem Cells
Source: PLoS One. 2015 Feb 23;10(2):e0118188. doi: 10.1371/journal.pone.0118188 (PMC4338147; doi:10.1371/journal.pone.0118188)

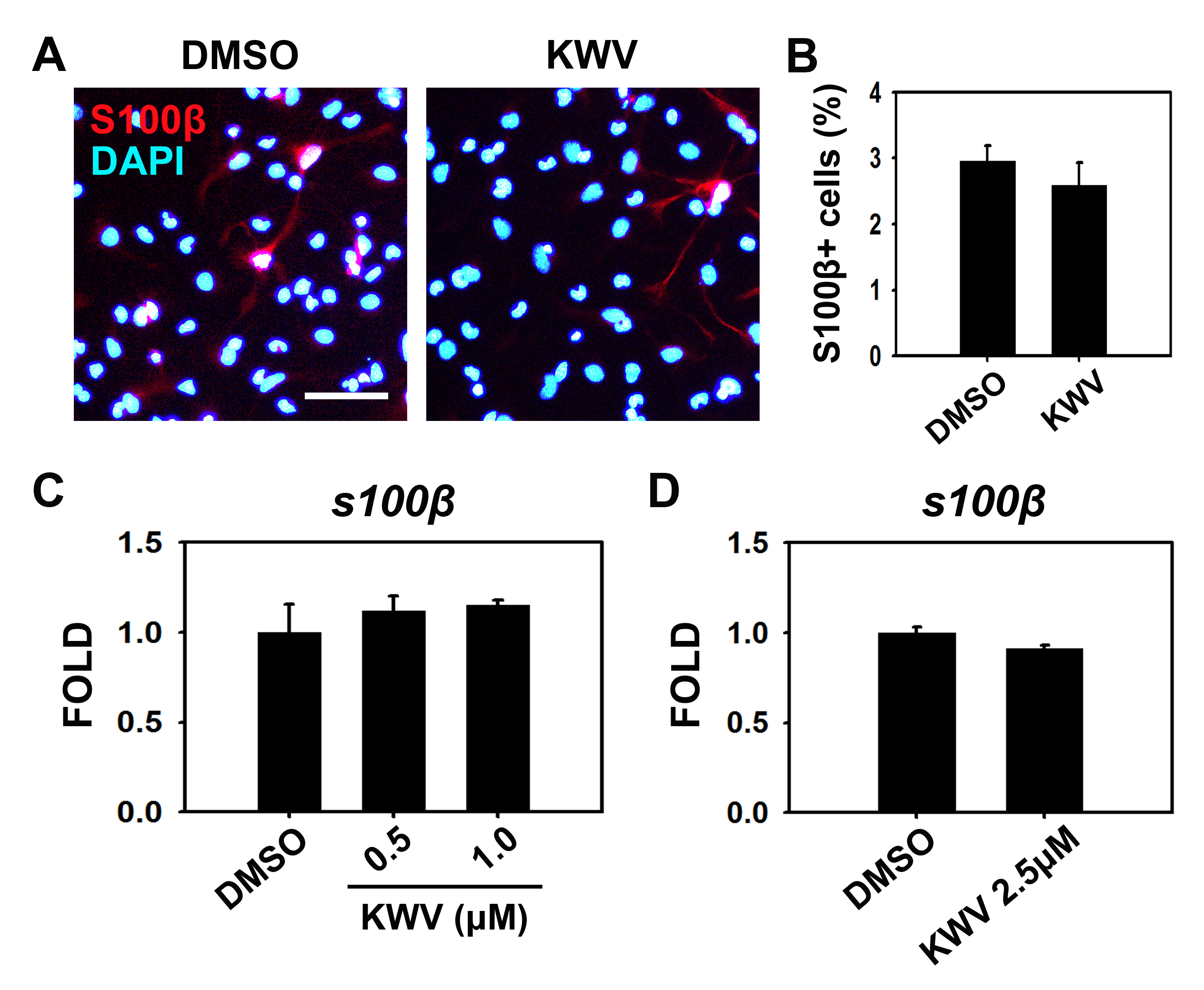

Supplement: S1 Fig — NSCs were expanded for 1 week, dissociated and plated for additional 4 days in the absence of growth factors and in the presence of KWV to induce differentiation. After fixation, cells were immunostained with anti-S100β antibody. (A) Representative images of cells treated with DMSO or KWV (0.5 μM) [S100β-positive cells (red), nuclei (blue)]. Scale bar, 50 μm. (B) Quantification of S100β-positive cells. The S100β-positive cells were counted and divided by the total number of nuclei. Data are presented as mean ± SD (n = 3). NSCs were expanded for 1 week, dissociated and plated for additional 2 days in the absence of growth factors and in the presence of DMSO or KWV. Total mRNA was extracted and RT PCR was performed. (C) mRNA expression levels of s100β in either DMSO or KWV (0.5 or 1.0 μM) treated cells. (D) mRNA expression levels of s100β in the presence of mitogens for 8 days (after 1 week of expansion, NSCs were dissociated and re-plated in the presence of mitogen and KWV for 1 day). Data are presented as the mean ± SD (n = 3). Statistical analysis was performed using the Student’s t-test. (TIF) [file pone.0118188.s001.tif]

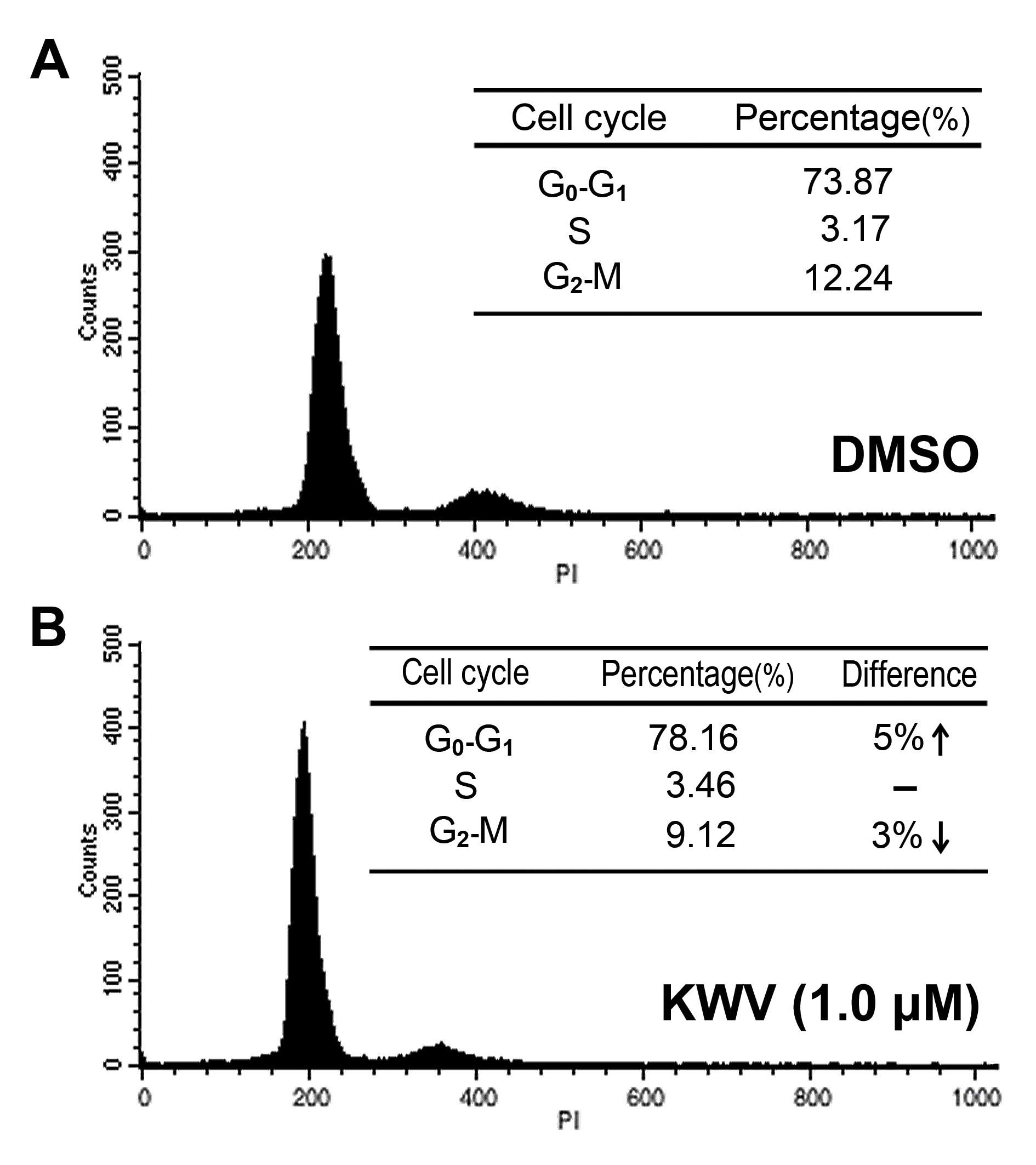

Supplement: S2 Fig — NSCs were treated with DMSO (A) and 1.0 μM KWV (B) for 2 days in the presence of EGF and FGF2 and analyzed using the FACS system to determine the cell cycle. Among the three independent results, we are showing one representative data. (TIF) [file pone.0118188.s002.tif]

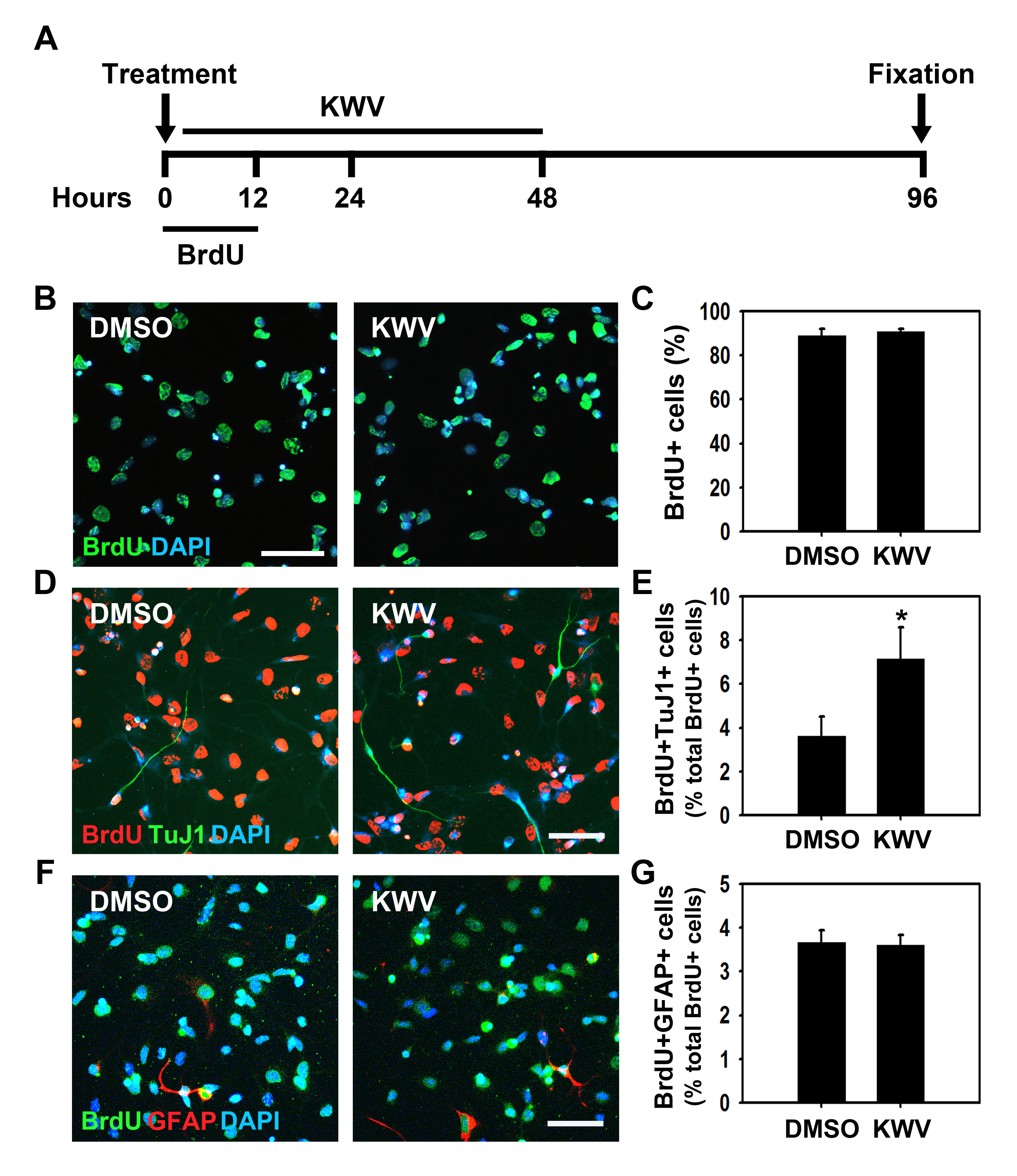

Supplement: S3 Fig — (A) Cells were treated with KWV (0.5 μM) for 2 days in the absence of EGF and FGF2. BrdU (10.0 μM) was added to the media during the first 12 h. (B) Representative immunofluorescence images of BrdU-positive cells (green) and nuclei (blue). (C) Quantification of BrdU-positive cells. (D) Representative photos of BrdU-positive cells (red), TuJ1-positive cells (green) and nuclei (blue). (E) Quantification of BrdU-positive and TuJ1-positive cells in DMSO or KWV (0.5 μM) treated cells. (F) Representative immunofluorescence images of BrdU-positive cells (green), GFAP-positive cells (red) and nuclei (blue) in DMSO or KWV treated cells. (G) Quantification of BrdU-positive and GFAP-positive astrocytes in DMSO or KWV treated cells. Scale bars, 50 μm (B, D, F). All cell count data were expressed as mean ± SD (n = 3). Statistical analysis was performed using the Student’s t-test (*P < 0.05). (TIF) [file pone.0118188.s003.tif]
