# Supplementary material for: Kuwanon V Inhibits Proliferation, Promotes Cell Survival and Increases Neurogenesis of Neural Stem Cells
Source: PLoS One. 2015 Feb 23;10(2):e0118188. doi: 10.1371/journal.pone.0118188 (PMC4338147; doi:10.1371/journal.pone.0118188)
Supplement: S1 Table — (DOCX) [file pone.0118188.s004.docx]

**S1 Table. Real-time PCR primers.**

| **Gene** | **Forward primer** | **Reverse primer** |
| --- | --- | --- |
| ***βIII tubulin*** | agccctctacgacatctgct | attgagctgaccagggaatc |
| ***p21*** | ctgctctcccttcctcagac | tgaggtaggaccaggaaacc |
| ***p27*** | ggaggaagatgtcaaacgtg | caagtcccgggttagttctt |
| ***bax*** | tggttgcccttttctactttg | gaagtaggaaaggaggccatc |
| ***bcl2*** | tgacttctctcgtcgctacc | gaactcaaagaaggccacaa |
| ***notch1*** | acttggctgcccgatactct | tggaagacaccctgagcatc |
| ***hes1*** | gaaagatagctcccggcatt | gtcacctcgttcatgcactc |
| ***gfap*** | agcggctctgagagagattc | agcaacgtctgtgaggtctg |
| ***s100*** | ggtgacaagcacaagctgaa | ctggaagtcacactccccat |
| ***neuroD*** | cccagcttaatgccatcttt | aaagggctgccttctgtaaa |
| ***gapdh*** | agttcaacggcacagtcaag | gtggtgaagacgccagtaga |
